# New Drugs, Appliances, and Things Medical

**Published:** 1889-11-30

**Authors:** 


### NEW DRUGS, APPLIANCES AND THINGS MEDICAL.

[All preparations, appliances, novelties, etc., of which a notice is desired, should be sent to The Editor, The Lodge, Porchester Square, W.]

### DIGBY'S CONCENTRATED SOUP BISCUIT.

On all hands science is ministering to the comfort, luxury, and convenience of man. Nowhere is this activity more apparent than in the production of portable food-stuffs. The latest brought to our notice is Digby's concentrated complete soup biscuit, which is said to contain fourteen different ingredients, including meat, cooked and pressed into biscuits, each and all of these ingredients being prepared in a perfectly dry state and formed into biscuit form by intense hydraulic pressure; thus it will keep in any climate without deteriorating. It is claimed that the article thus prepared will keep in any climate, and the soup can be ready for use in a few minutes. For the Army and Navy, and the masses generally, in short, wherever portability is of importance, this biscuit will prove invaluable. We have tried the soup, and find it easily made, most palatable, and hygienic. It is a capital thing to keep in the kitchen for making stock. The inventor states that a 1lb. biscuit will make five quarts of nourishing soup, can be sold retail for 4d., and is enough, with bread, to make a meal for several people. A convenient soup powder is also made by the same firm.

### LISTER'S NEW ANTISEPTIC DRESSING.

One of the most important additions to the methods of antiseptic surgery that has been made for many years was lately announced by Sir Joseph Lister, at a meeting of the Medical Society. He first described the various ways in which he arrived, by slow and devious routes, at his discovery. Of the vast amount of labour which these investigations have involved, none but the author can have any adequate idea. Briefly stated, the new antiseptic is a double salt of cyanide of mercury and zinc, in close combination with starch. The antiseptic does not wash out from gauze charged with the prepared starch, so that the dressing remains perfect in spite of any amount of discharge. The other two great advantages are that it is a trustworthy antiseptic and is unirritating. In practice, the gauze next the wound is washed out with a one in twenty solution of carbolic acid. Professor Lister rarely speaks, and this announcement will be hailed with delight by all true believers in his theory. Methods have been and are admittedly imperfect, a fact which gives the advocates of cleanliness *versus* antiseptics their chief standing ground. To his disciples the Professor offers his dressing as "the most satisfactory that I have hitherto met with; and I venture to hope that you will regard it as a not unwelcome addition to your resources."

